# In Situ Oxidation of Cu_2_O Crystal for Electrochemical Detection of Glucose

**DOI:** 10.3390/s19132926

**Published:** 2019-07-02

**Authors:** Chenlin Lu, Zhipeng Li, Liwei Ren, Nan Su, Diannan Lu, Zheng Liu

**Affiliations:** 1 State Key Lab of Chemical Engineering, Ministry of Science and Technology, Beijing 100084, China; 2 Department of Chemical Engineering, Tsinghua University, Beijing 100084, China; 3 College of Biological and Pharmaceutical Sciences, China Three Gorges University, Yichang 443002, China

**Keywords:** glucose detection, glucose sensor, electrochemical analysis, Cu_2_O nanoparticles, electrochemical deposition

## Abstract

The development of a sensitive, quick-responding, and robust glucose sensor is consistently pursued for use in numerous applications. Here, we propose a new method for preparing a Cu_2_O electrode for the electrochemical detection of glucose concentration. The Cu_2_O glucose electrode was prepared by in situ electrical oxidation in an alkaline solution, in which Cu_2_O nanoparticles were deposited on the electrode surface to form a thin film, followed by the growth of Cu(OH)_2_ nanorods or nanotubes. The morphology and electrocatalytic activity of a Cu_2_O glucose electrode can be tuned by the current density, reaction time, and NaOH concentration. The results from XRD, SEM, and a Raman spectrum show that the electrode surface was coated with cubic Cu_2_O nanoparticles with diameters ranging from 50 to 150 nm. The electrode exhibited a detection limit of 0.0275 mM, a peak sensitivity of 2524.9 μA·cm^−2^·mM^−1^, and a linear response range from 0.1 to 1 mM. The presence of high concentrations of ascorbic acid, uric acid, dopamine and lactose appeared to have no effects on the detection of glucose, indicating a high specificity and robustness of this electrode.

## 1. Introduction

Monitoring the concentration of glucose is essential for diabetes diagnosis [1], fermentation [2], chemical synthesis [3], and other industrial applications. In recent years, continuous effort has been made to develop electrochemical [4] and other methods including optical [5], acoustic [1], chemiluminescence [6], fluorescent nanogels [7], and near-infrared spectroscopy [8], for glucose detection. The electrochemical method is one with proven advantages in terms of accuracy, sensitivity, ease of operation, and portability. In 1962, Clark and Lyons proposed the concept of the glucose enzyme electrode and prepared the first glucose electrode [9]. In order to avoid the interference of background O_2_ in the sample, electron acceptors such as ferrocene derivatives are used to specify the electron transfer in GOx recycling [4,10]. More recently, a virgin electrode without mediators was used to regenerate GOx (flavin adenine dinucleotide) directly on the surface of the electrodes [11,12,13]. While enzymatic sensors are routinely used in blood glucose detection, their applications to on-site and in situ detection, as often requested by research and engineering practice, are hindered by the limited stability of GOx and unsatisfactory sensitivity due mainly to deficient electron transfer. To overcome these weaknesses, non-enzymatic glucose sensors [14] that directly oxidize glucose at the surface electrode are pursued, and numerous materials have been developed, such as palladium nanoparticles coated functional carbon nanotubes [15], Ti/TiO_2_ nanotube arrays/Ni composites [16], electrospun palladium (IV)-doped copper oxide composites in the form of nanofibers [17], nanoporous Au [18], and Cu nanoclusters/multi-walled carbon nanotubes (MWCNT) composites [19]. 

Compared with Pt and Au, Ni and Cu exhibit higher efficiency in the electrochemical detection of glucose because of the formation of Ni(II)/Ni(III)and Cu(II)/Cu(III) redox pairs [20,21]. CuO-based glucose sensors are also attractive due to their rich arability and low toxicity [22]. The oxidation of glucose on a CuO-based glucose electrode has not been fully elucidated [23,24]; however, it is suggested that Cu(II) forms Cu(III), which is capable of oxidizing glucose to gluconolactone and is reduced to Cu(II) after the glucose oxidation. The conventional method to fabricate a CuO glucose electrode consists of several steps, in which the first step is the hydrothermal synthesis of CuO nanoparticles, typically at approximately 100 °C [25,26]. The resulting CuO nanoparticles are then mixed with eutectic polymer binders, such as Nafion, Polyvinylidene fluoride (PVDF) and chitosan, forming an “ink”. Finally, the ink is coated onto the virgin noble electrode to form a thin layer [25,27,28]. The sensitivity of such an electrode is affected by the presence of binders with no oxidation capabilities. 

Although nanomaterials such as Carbon nanotubes (CNT) and graphene are blended with CuO nanoparticles [28] giving an improved conductivity, a direct contact of CuO with glucose and an accelerated electron transfer remains desirable for improved sensitivity and responsive speed. In situ fabrication methods have been explored in previous studies by chemical or electrochemical oxidation under different conditions [29,30,31,32]. Here, we present a new method for an in situ fabrication of a Cu_2_O-based glucose electrode, in which a Cu_2_O electrode was prepared by electrooxidation of metallic copper as an anode in alkaline solution, forming a Cu_2_O layer which consists of particles with diameters of 30–150 nm on the surface of the Cu electrode. We expect the circumvention of the “ink” not only simplifies the preparation process but also, and more importantly, allows direct contact of glucose with the electrode and facilitates the electron transport, thereby promising an improved sensitivity and responsiveness. The sensitivity and specificity of the detection, as well as the stability of the electrode, were examined. The effects of the preparation conditions on the morphology and electrocatalytic activity of a Cu_2_O glucose electrode were investigated to understand the fabrication mechanism of high catalytic activity Cu_2_O nonenzymic glucose sensors. The results reveal that the morphology and crystal phase are determined by the ratio of current density and NaOH concentration (J/c). As J/c increases, the morphology changes from Cu_2_O nanoparticles to Cu(OH)_2_ nanotubes. Cu_2_O nanoparticles are deposited on the electrode surface, forming a thin film, followed by the growth of Cu(OH)_2_ nanorods or nanotubes; among them, Cu_2_O nanoparticles exhibit higher activity. These results provide a rapid and efficient method, using mild conditions, for the in situ synthesis of a Cu_2_O electrode with good stability and repeatability for electrochemical detection of glucose.

## 2. Materials and Methods

### 2.1. Materials and Reagents

A Cu electrode, Ag/AgCl electrode, and Pt electrode were purchased from Aidahengsheng, Tianjin, China. Ascorbic acid (99%, analytical pure) was purchased from Solarbio, Beijing, China. Lactose (99%, analytical pure) was purchased from Sinopharm Chemical Reagent, Beijing, China. Dopamine (99%, analytical pure) and uric acid (99%, analytical pure) were purchased from Aladdin, Shanghai, China. All agents were used without further purification. 

### 2.2. Preparation of the Cu_2_O Electrode

The Cu_2_O electrode was synthesized via an in situ electrical oxidation method in alkaline solution. First, a disk Cu electrode with a diameter of 3 mm was polished using Al_2_O_3_ powders of 300 nm and 50 nm in diameter, respectively. After washing in ultrasonic baths of ethanol and distilled water consecutively and drying in a N_2_ atmosphere, the Cu electrode was implemented as the working electrode on CHI 852D (CH Instruments, Shanghai, China) with a 3-electrode system, in which the counter electrode was a Pt electrode and the reference electrode was Ag/AgCl, and oxidized for 300 s at a constant current density of 1.415 mA/cm^2^ (0.1 mA for a ϕ3 disk electrode) in 3 M NaOH solution. The obtained Cu_2_O electrode was washed with distilled water and dried in a N_2_ atmosphere.

### 2.3. Characterization Apparatus 

A field-emission scanning electron microscope (FESEM) (Merlin, Carl Zeiss Jena, Germany) was used to observe the microstructures of Cu_2_O glucose electrode. The applied voltage was 15 kV. The crystalline phase of the structures was identified by X-ray diffraction (S2, Rigaku, Tokyo, Japan) with Cu Kα radiation. Raman spectroscopy was performed with a Raman microscopy system (LabRAM HR, HORIBA Jobin Yvon, Paris, France). A YAG laser served as an excitation source, and the applied wavelength was 532 nm.

### 2.4. Electrochemical Measurements

Electrochemical tests including cyclic voltammetry (CV) and Amperometric (*i*–*t*) measurements were conducted on CHI 852D (CH Instruments, Shanghai, China) with a 3-electrode system, in which the working electrode, the counter electrode, and the reference electrode were the Cu_2_O electrode synthesized as mentioned in Section 2.2, Pt electrode and Ag/AgCl (saturated) electrode, respectively. 

## 3. Results and Discussion

### 3.1. Characterization of the Cu_2_O Glucose Electrode

**Figure 1:**
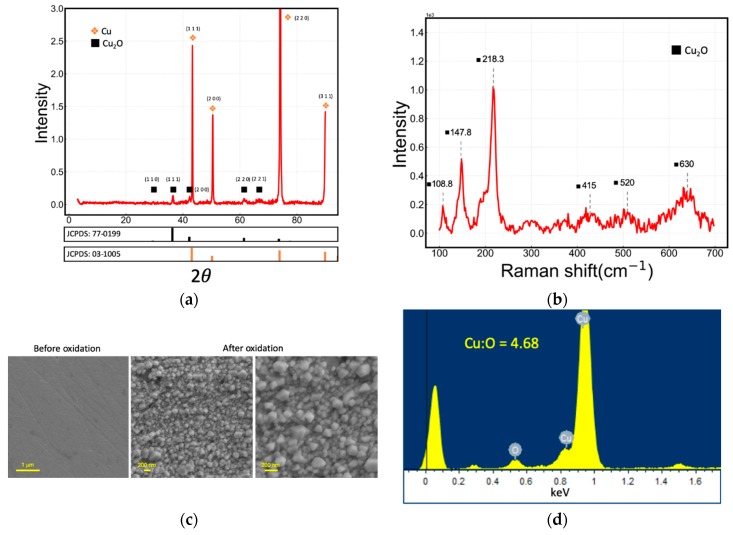
(**a**) XRD pattern, (**b**) Raman spectrum, (**c**) SEM images of the newly in situ fabricated Cu_2_O electrode, and (**d**) energy-dispersive X-ray spectroscopy (EDS) analysis.

The as-prepared Cu_2_O glucose electrode was characterized by XRD, SEM and a Raman spectrum, as shown in Figure 1. 

From Figure 1a, it is interpreted that the major crystal phase of the Cu_2_O glucose electrode is the cubic phase of Cu_2_O (JCPDS card no.77-0199, Fm3m, *a*_0_ = *b*_0_ = *c*_0_ = 4.258 Å). The copper metal substrate (JCPDS card no.03-1005, Fm3, *a*_0_ = *b*_0_ = *c*_0_ = 3.6077 Å) is also present. Figure 1b shows the Raman spectrum of the Cu_2_O glucose electrode. The characteristic peaks at 108.8, 148.7, 218.3, 415, 520 and 630 cm^−1^ demonstrate the formation of Cu_2_O on the Cu electrode. It can be seen from Figure 1c that cubic nanoparticles with diameters of 30–150 nm are dispersed evenly on the electrode surface. As can be seen from Figure 1d, only Cu and O are displayed in the energy-dispersive X-ray spectroscopy (EDS) analysis; it is concluded that the cubic phase crystals on the Cu electrode are Cu_2_O, as described elsewhere [23,33,34]. 

### 3.2. Electrochemical Behavior of the Cu_2_O Electrode

**Figure 2:**
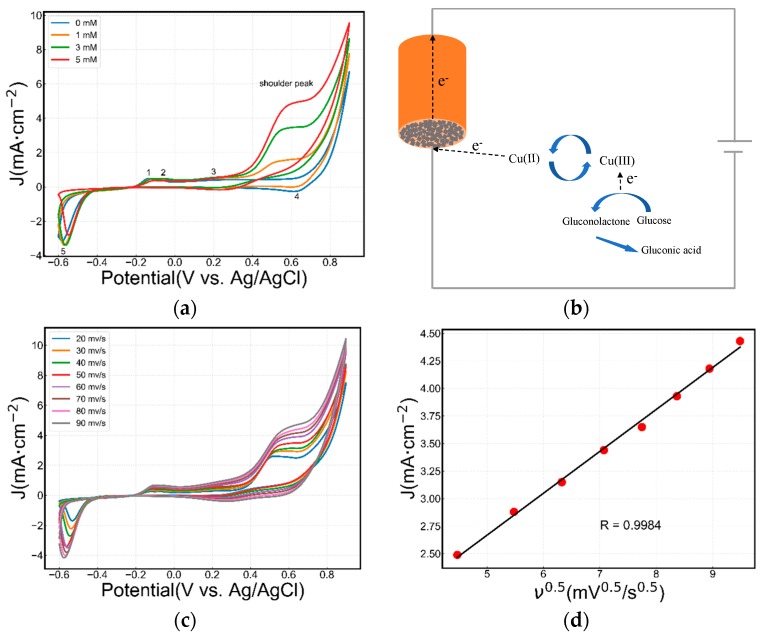
(**a**) Cyclic voltammograms (cathodic sweep first) of a Cu_2_O electrode in 50 mM NaOH electrolyte. Scan rate: 50 mV/s. (**b**) Mechanism of glucose oxidation on the Cu_2_O electrode. (**c**) Cyclic voltammograms (CVs) of the Cu_2_O electrode at scan rates varying from 10–90 mV/s in 50 mM NaOH solution containing 3 mM glucose. (**d**) Linear regression of the peak current and square root of the scan rate.

Figure 2a shows the cyclic voltammetry of the Cu_2_O electrode prepared in a 50 mM NaOH solution at a scan rate of 50 mV/s, in the absence or presence of glucose. Here, five distinctive redox peaks can be observed on the Cu_2_O electrode. Peak 1 is assigned to the transition of Cu(0)/Cu(I), while peak 2 includes two transitions of Cu(0)/Cu(II) and Cu(I)/Cu(II). Peak 3 is related to the transition of Cu(II)/Cu(III) in alkaline solution [27]. Peak 4 and peak 5 are related to the transitions of Cu(III)/Cu(II) and Cu(II)/Cu(I), respectively [24]. In the presence of glucose, the CV profile also contains peak 1 and peak 2, while peak 3 disappears and is replaced by a shoulder peak around 0.5 V (vs. Ag/AgCl). The height of this peak is related to the glucose concentration.

Marioli and Kuwana [35] and Chen et al. [36] have attributed the difference in the cyclic voltammograms obtained in the absence and presence of glucose to the transition of Cu(II)/Cu(III). First, glucose turns to an enol form upon isomerization in an alkaline medium, then, the active intermediate is oxidized to gluconolactone by Cu(III) followed by the hydrolysis of gluconolactone and forms gluconic acid. The above-mentioned process is shown in Figure 2b.

Cyclic voltammetry curves were performed at different scan rates of 20–90 mV/s in the presence of 3 mM glucose and 50 mM NaOH solution, as shown in Figure 2c,d. It is shown that the intensity of the shoulder peak near 0.5 V (vs. Ag/AgCl) increases as the scan rate increases. Moreover, a linear relationship between current intensities of the peak at 0.5 V and the square root of scan rates is observed (Figure 2d). According to the Randles–Sevcik equation, good fit linearity reflects a sufficiently rapid electron transfer reaction rate, indicating the diffusion-controlled progression.

### 3.3. Amperometric Detection of Glucose

**Figure 3:**
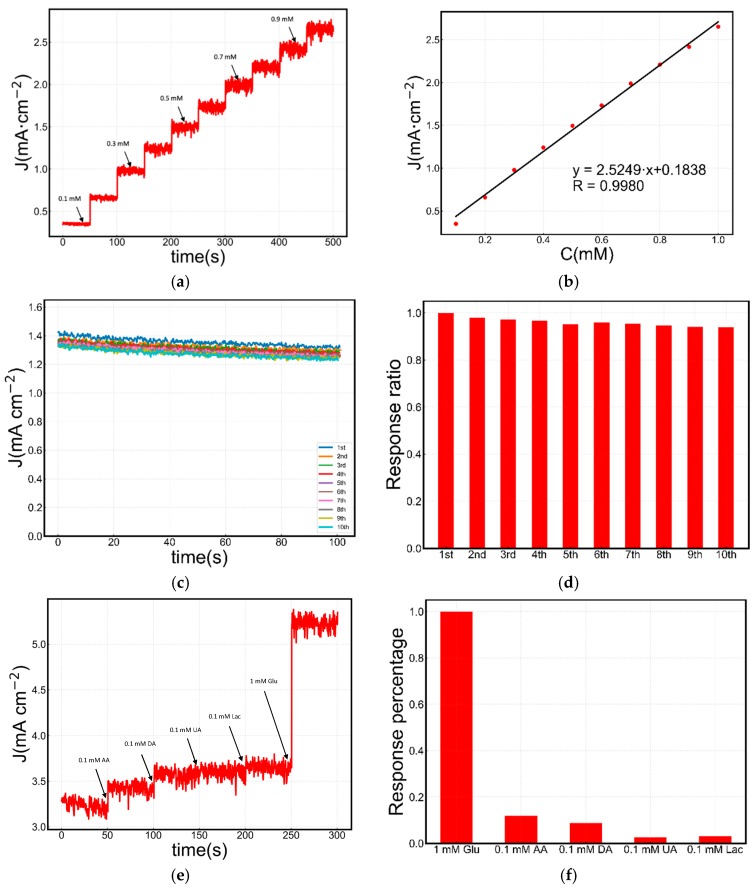
(**a**) Amperometric *J–t* test with successive addition of glucose at 0.5 V (vs. Ag/AgCl). (**b**) The calibration curve of (**a**). (**c**) Amperometric *J–t* tests repeated 10 times in 50 mM NaOH solution containing 1 mM glucose. (**d**) Current response percentage of each amperometric *J–t* test in (**c**). (**e**) Amperometric responses of the Cu_2_O electrode to the successive dropwise additions of interfering species (ascorbic acid (AA), dopamine (DA), uric acid (UA), lactose (Lac)) and glucose. (**f**) Current response percentage of (**e**).

We then determined the current density at different glucose concentrations in 50 mM NaOH solution and obtained the results shown in Figure 3.

The amperometric *J–t* curve in Figure 3a shows that the current density responds to the change of glucose concentrations quickly. The current becomes stable within 3 s after each injection of fresh glucose solution. Figure 3b shows an almost ideal linear response of the current intensity within the glucose concentration range from 0.1 to 1.0 mM. The working voltage is 0.5 V vs. Ag/AgCl, which is higher than the standard potential of the oxygen evolution reaction (0.204 V vs. Ag/AgCl). Therefore, small oxygen bubbles accumulated on the surface of the electrode at a very slow rate. The unreleased small bubbles result in increased noise.

Figure 3b shows the linear dependency of the current response versus different glucose concentrations. The sensitivity of this Cu_2_O electrode is 2524.9 μA·cm^−2^·mM^−1^, when glucose concentrations are in the range of 0.1 to 1 mM. The limit of detection (LOD) of the Cu_2_O electrode is 2.57 µM, which is calculated by substituting the standard deviation of the background signal with the mean standard deviation of the fitting results (S/N = 3).

### 3.4. Stability and Selectivity

Figure 3c,d show that the current density decrease is only 6.09% after ten cycles. The recycle is critical for practical applications, and such a recycle time of Cu_2_O has not yet been reported before. The selectivity and specificity of the Cu_2_O glucose electrode were examined by adding other species in considerable concentrations. The current response was recorded in 50 mM NaOH solution with successive injection of 0.1 mM ascorbic acid (AA), 0.1 mM dopamine (DA), 0.1 mM uric acid (UA), 0.1 mM lactose (Lac) and 1 mM glucose (Glu), as shown in Figure 3e,f, from which it is concluded that these interferents have little effect on the detection of glucose since their physiological concentrations are AA (30 μM) [37], DA (0.14 nM) [38], UA (300 μM) [39] and Lac (1.5 μM) [40], respectively. The prepared Cu_2_O electrode can detect glucose in the presence of other traditional contaminants in their physiological concentrations.

Table 1 details the Cu_2_O electrodes and other metal-based electrodes utilized under alkaline conditions reported elsewhere, as well as their performance in terms of sensitivity, linear response range, detection limit, response time and cycles times. We profiled the data listed in Table 1, as shown in Figure S1. It can be concluded that the Cu_2_O electrode developed in the present study is superior in terms of both sensitivity and durability with regard to recycle times. The high sensitivity, being close to Cu_2_O/MoS_2_, we believe, is attributed to the circumvention of polymer binder, which leads to direct electron transport, as evidenced by the reduction of the overpotential [27]. The response time is a function of the thickness of the Cu_2_O layer and can be tuned by the current density and electro-oxidation time. This is advantageous for manipulating the structure of the electrode to meet the given requirement of sensitivity and response time. The electrode developed by the present study has the highest durability in terms of cycles times, which, we believe, benefits from the in situ generation of Cu_2_O nanostructures on the surface of the metallic copper electrode. Such high durability makes it promising for long-term detection.

### 3.5. Mechanism of In Situ Deposition of Cu_2_O

**Figure 4:**
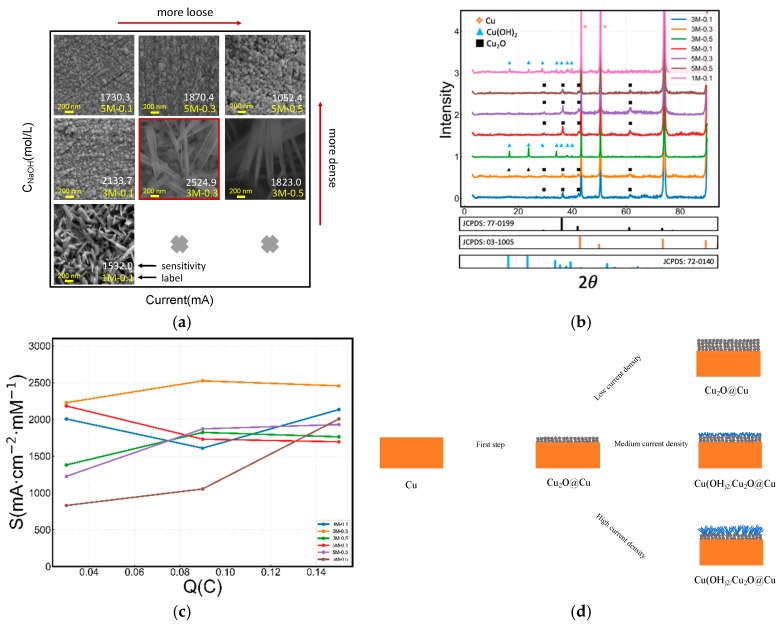
(**a**) SEM images. (**b**) XRD patterns of sensors prepared in different NaOH concentrations and current intensities. (**c**) The relationship between sensitivity and charge consumption during preparation for the Cu_2_O electrodes fabricated under different conditions. (**d**) Crystal growth mechanism of electro-oxidation of Cu as an anode under alkaline conditions.

We also examined the effect of the fabrication conditions on the microstructure and electrochemical activity. The morphology and responsive behavior of the yielded Cu_2_O electrodes were determined, respectively. As shown in Figure 4a, their fabrication conditions were used to label the electrodes. For instance, 1M-0.1-100 indicates that the Cu_2_O electrode is fabricated in a condition of 1.0 M NaOH, 0.1 mA current and 100 s electrooxidation time. The SEM images of the 3M-0.1-300, 3M-0.1-1200, 5M-0.1-300 and 5M-0.1-1200 electrode (Figure S2) reveal that the electrooxidation time has little effect on the morphology of the electrodes. Therefore, we fixed the electro-oxidation time as 300 s for further discussion. To clarify, only the concentration of NaOH and the current density are labelled to indicate samples, as shown in Figure 4a.

Figure 4a shows the SEM images of the Cu_2_O electrodes fabricated under different conditions. The electrodes were characterized by XRD in Figure 4b and Raman in Figure S3. The main crystal phase of nanoparticles on 3M-0.1, 3M-0.3, 5M-0.1, 5M-0.3 and 5M-0.5 electrodes is face-centered cubic Cu_2_O (JCPDS card no.77-0199, Fm3m, $a_{0}=b_{0}=c_{0}=4.258$ Å), while the main crystal phase of the nanorod or nanotube on 3M-0.5 and 1M-0.1 electrodes is orthorhombic Cu(OH)_2_ (JCPDS card no.72-0140, Cmcm, Å, Å and Å).

The current density and the NaOH concentration may also significantly affect the morphology of the Cu_2_O electrode. It is observed that the current intensity and the NaOH concentration significantly affect the morphology of the Cu_2_O electrode. Here, we establish a model to elucidate this interesting phenomenon. The current density, which is denoted as J, is equivalent to the rate of Cu^+^/Cu^2+^ formation during the electro-oxidation reaction. The concentration of NaOH, which is denoted as c, is related to the rate of consuming Cu^+^ and Cu^2+^. In the case of a low value of J/c, the Cu^2+^ or Cu^+^, produced by electro-oxidation is immediately consumed to form nanoparticles due to excessive OH^−^ at the surface of the electrode. This prevents further nucleation and results in small polyhedral nanoparticles depositing on the surface of electrode, as shown in Figure 4a (e.g., 5M-0.1, 5M-0.3, 5M-0.5, and 3M-0.1). The polyhedral nanoparticles are face-centered cubic Cu_2_O. When the value of I/c increases, the rate of Cu^+^/Cu^2+^ formation gradually reaches that of Cu^+^/Cu^2+^ consumption, resulting in the formation of incomplete nanotubes (akin to a nanosheet), as shown in Figure 4a (e.g., 3M-0.3-300). If we further increase the value I/c by increasing the current intensity or reducing the concentration of NaOH, the intact nanotubes composed of Cu(OH)_2_ form on the surface of the Cu electrode (e.g., 3M-0.5-300), as shown in Figure 4a. According to the above discussion, the mechanism of forming different Cu_2_O nanostructures is illustrated in Figure 4d.

The oxidation time is vital for catalytic activities by determining the depth of the active material, which also relates to mass transfer resistance during the glucose oxidation reaction [61,62,63]. Figure 4c shows the relationship between sensitivity and charge consumption for each electrode. According to the similar catalytic activity at the optimum oxidation time and the fabricating mechanism shown in Figure 4d, the first step is the formation of the Cu_2_O film, followed by the reaction between Cu_2_O and OH^−^ to produce Cu(OH)_2_ nanorods or nanotubes. As a result, the electrodes prepared under different conditions contain a layer of Cu_2_O film. It could be inferred that cubic Cu_2_O exhibits a higher catalytic activity than orthorhombic Cu(OH)_2_ and the peak sensitivity is 2524.9 μA·cm^−2^·mM^−1^.

## 4. Conclusions

In this paper, Cu_2_O electrodes for glucose detection were prepared in situ by an electrochemical oxidation method. The electrode exhibits a high sensitivity of 2524.9 μA·cm^−2^·mM^−1^ for glucose detection, with a linear response range of 0.1–1 mM and a detection limit of 0.0275 mM. Moreover, the electrode showed good stability and repeatability. The electrodes fabricated in situ under different conditions were characterized by SEM, XRD, and a Raman spectrum. The mechanism relating preparation condition, morphology and catalytic activity was clarified. Here, we describe a promising method and underlying mechanism to fabricate cubic Cu_2_O electrodes with reduced resistance and overpotential but high sensitivity. Furthermore, the advantages of high repeatability, simple operation and low energy consumption make the described method possible for practical application.

## Figures and Tables

**Table 1:** Comparison of different glucose sensors performance.

Electrode	Sensitivity (μA·mM^−1^·cm^−2^)	Linear Range (mM)	Detection Limit (μM)	Response Time (s)	Cycles (times)	Ref.
Octahedral Cu_2_O	293.893	0.1–5	5.11	5	7	[41]
Cu_2_O/graphene	1330.05	0.01–3.0	0.36	7	7	[42]
Cu_2_O/MoS_2_	3108.87	0.01–4.0	1	3	10	[43]
CQDs/O-Cu_2_O	298	0.02–4.3	8.4	10	3	[44]
Cu_2_O/TiO_2_ nanotube	14.56	3.0–9.0	62	3	5	[45]
rGOs wrapped Cu_2_O	285	0.3–3.3	3.3	9	1	[46]
Hollow Cu_2_O	2038.2	0.00125–0.0375	0.41	3	5	[47]
Cu/Cu_2_O/CS	63.8	0.01–0.69	5	5	1	[48]
Cu_2_O/AlOOH/rGO	155.1	0.005–14.77	2.6	5	3	[49]
rGOs-porous Cu_2_O	185.1	0.01–6	0.05	3	1	[50]
Au@Cu_2_O	715	0.05–2.0	18	20	5	[23]
polyhedral Cu_2_O	300.96	1.2–298	0.144	4	1	[51]
Cu_2_O/PC platinum	507	0.1–2.5	26	5	3	[52]
DH Cu_2_O/GCE	1231.7	0.019–1.089	18.5	3	2	[53]
Nafion/Cu@Cu_2_O/GCE	1420	0.0007–2.0	40 nm	2	7	[27]
Cu_2_O NPs/G_3_DN/CP	2310	0.00048–1.813	0.14	1.6	4	[54]
Cu_2_O/Cu	62.29	0.05–6.75	37	5	2	[31]
Cu_2_O/Cu	728.67	0.01–7.53	3	3.6	1	[30]
CuO/Cu	761.9	0.002–20	2	1	7	[32]
CuO/CuOx/Cu	1890	0.002–15	0.05	1	6	[29]
Co_3_O_4_/GOH/GCE	492.8	0.25–10	-	8	1	[55]
Co_3_O_4_/rGO/GCE	1366	0.0005–1.277	0.18	2	2	[56]
Co_3_O_4_/rGOP	1.21	0.04–4	1.4	-	1	[57]
Ni(OH)_2_/insulin/rGO/Au	18.9	0.005–10	5	7	2	[58]
NiO/rGO/GCE	1138	0.001–0.4	0.18	2	10	[59]
NiO/DNA/graphene/GCE	9	0.001–8	2.5	8	10	[60]
Cu_2_O@Cu	2524.9	0.1–1.0	2.57	3	10	This work

## Supplementary Materials

The following are available online at https://www.mdpi.com/1424-8220/19/13/2926/s1, Figure S1: Comparison of different glucose sensors’ performance, Figure S2: The SEM images of 5M-0.1-300, 5M-0.1-1200, 3M-0.1-300, 5M-0.1-1200, Figure S3: The Raman spectrum of the Cu_2_O electrodes fabricated under different conditions.
