# Supplementary material for: In Situ Oxidation of Cu2O Crystal for Electrochemical Detection of Glucose
Source: Sensors (Basel). 2019 Jul 2;19(13):2926. doi: 10.3390/s19132926 (PMC6651079; doi:10.3390/s19132926)
Supplement: Supplementary file 1 [file sensors-19-02926-s001.pdf]

Supporting Information

In Situ Oxidation of Cu_2O Crystal for Electrochemical Detection of Glucose

Chenlin Lu, Zhipeng Li, Liwei Ren, Nan Su, Diannan Lu and Zheng Liu

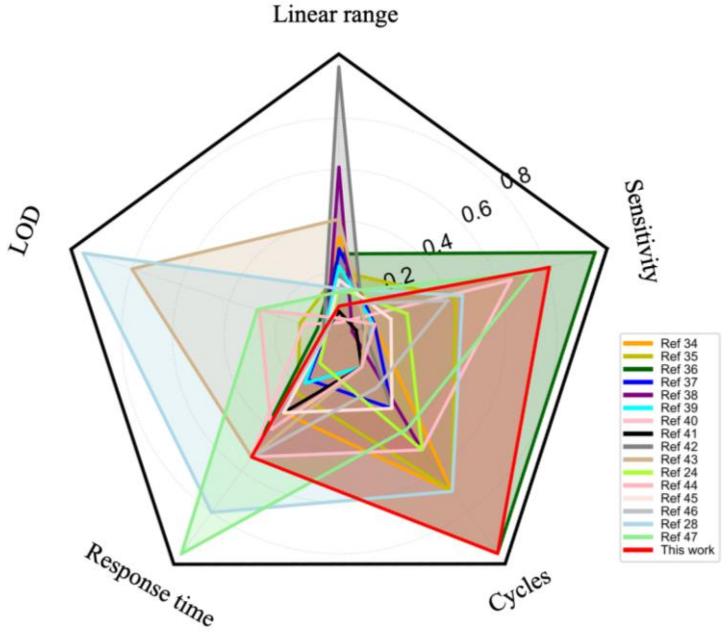

Figure S1. Comparison of different glucose sensors performance.

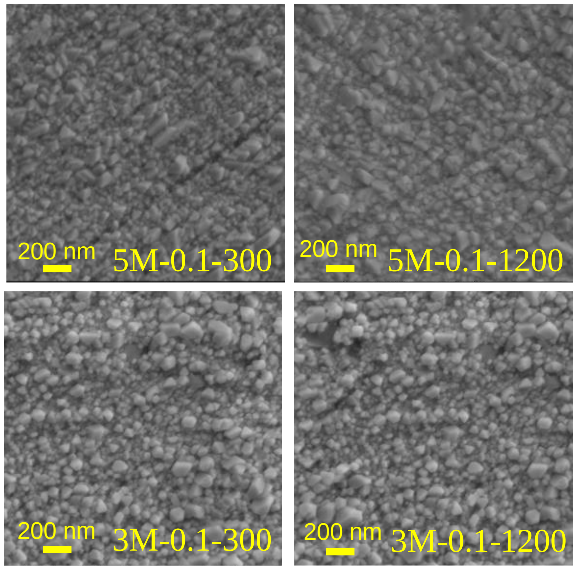

Figure S2. The SEM images of 5M-0.1-300, 5M-0.1-1200, 3M-0.1-300, 5M-0.1-1200.

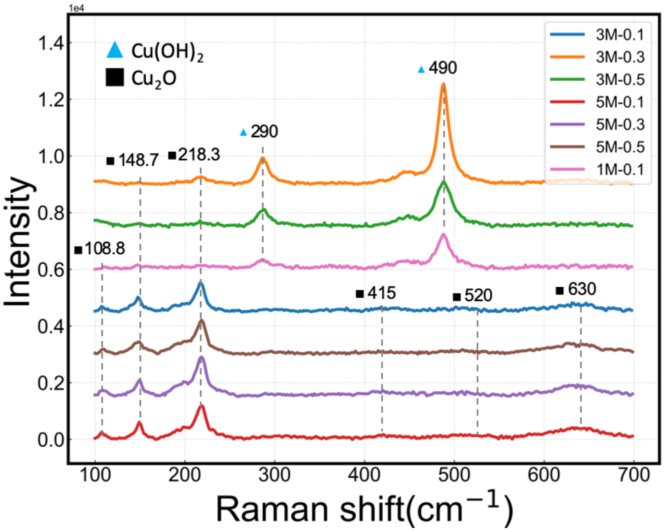

Figure S3. The Raman spectrum of the Cu_2O electrodes fabricated under different conditions.

The Raman spectra of 3M-0.1, 3M-0.3, 5M-0.1, 5M-0.3 and 5M-0.5 electrodes exhibit characteristic peaks at 108.8 cm^{-1}, 148.7 cm^{-1}, 218.3 cm^{-1}, 520 cm^{-1} and 630 cm^{-1}, which are attributed to Cu_2O [1,2]. The Raman spectra of 3M-0.5, 3M-0.3 and 1M-0.1 exhibit the characteristic peaks of $Cu(OH)_2$ at 290 cm^{-1} and 490 cm^{-1}, according to previous researches [2–4]. It should be noticed that the Raman spectrum of 3M-0.3 exhibits the both characteristic peaks of Cu_2O and $Cu(OH)_2$. This agrees with the SEM in Figure S1 that shows the existence of nanoparticles and nanotubes.

References

1. Chan, H.Y.H.; Takoudis, C.G.; Weaver, M.J. Oxide film formation and oxygen adsorption on copper in aqueous media as probed by surface-enhanced Raman spectroscopy. *J. Phys. Chem. B* **1999**, *103*, 357–365.

2. Deng, Y.; Handoko, A.D.; Du, Y.; Xi, S.; Yeo, B.S. In Situ Raman Spectroscopy of Copper and Copper Oxide Surfaces during Electrochemical Oxygen Evolution Reaction: Identification of CuIII Oxides as Catalytically Active Species. *ACS Catal.* **2016**, *6*, 2473–2481.

3. Hamilton, J.C.; Farmer, J.C.; Anderson, R.J. In situ Raman spectroscopy of anodic films formed on copper and silver in sodium hydroxide solution. *J. Electrochem. Soc.* **1986**, *133*, 739–745.

4. Reyter, D.; Odziemkowski, M.; Bélanger, D.; Roué, L. Electrochemically Activated Copper Electrodes Surface Characterization, Electrochemical Behavior, and Properties for the Electroreduction of Nitrate. *J. Electrochem. Soc.* **2007**, *154*, K36–K44.
